# Assessment of the Dutch Healthy Diet index 2015 in the Lifelines cohort study at baseline

**DOI:** 10.1038/s41430-023-01372-x

**Published:** 2023-11-28

**Authors:** A. Mireille Baart, Elske M. Brouwer-Brolsma, Hanne B. T. de Jong, Jeanne H. M. de Vries, Edith J. M. Feskens

**Affiliations:** Wageningen University & Research, Division of Human Nutrition and Health, Wageningen, The Netherlands

**Keywords:** Nutrition, Public health

## Abstract

**Background:**

Dietary indices are useful measures to investigate associations between dietary intake and disease development. The Dutch Healthy Diet index 2015 (DHD2015-index), a measure of diet quality, assesses adherence to the 2015 Dutch dietary guidelines. We assessed the DHD2015-index in the Lifelines cohort study, and compared calculations from basic and detailed dietary intake data. This article replaces the retracted article that was published on 16 May 2022 [1].

**Methods:**

Dietary intake was assessed with a specially developed Food Frequency Questionnaire (FFQ) called Flower-FFQ, which consists of one main questionnaire (heart-FFQ), which asks for intakes of major food groups, and three complementary questionnaires (petal-FFQs), which ask for detailed information on food types within major food groups of the heart-FFQ. The DHD2015-index was assessed using data from the total Flower-FFQ (for 56,982 participants), and using data from the heart-FFQ only (for 129,030 participants). Agreement between the two indices was assessed with correlation and cross-classification.

**Results:**

The median (25th–75th percentile) DHD2015-index score was 75 (65–85) for men and 81 (70–91) for women based on the Flower-FFQ, and 68 (58–77) for men and 73 (63–82) for women based on the heart-FFQ. The Kendall’s tau-b correlation coefficient between the two scores was 0.67 for men and 0.66 for women. Cross-classification into quartiles of the DHD2015-index showed that 59–60% of participants were classified in the same quartile, 36–37% in the adjacent, and 4% in the non-adjacent.

**Conclusion:**

Dietary data from the Flower-FFQ provide the most optimal information to assess the DHD2015-index. However, the DHD2015-index from the heart-FFQ showed good agreement with the index from the Flower-FFQ of ranking participants according to diet quality, and can be used when the DHD2015 index from the Flower-FFQ is not available.

## Introduction

Large epidemiological studies offer the opportunity to investigate associations between dietary intake and disease development [2]. The Lifelines cohort study is a Dutch multi-disciplinary prospective population-based cohort study, that was established in 2006 as a resource for international researchers, to obtain insight into the etiology of healthy ageing [3, 4]. The Lifelines database contains, among others, detailed dietary intake data, including intake of energy, macro- and micronutrients, and food groups [5], which were collected using a Food Frequency Questionnaire (FFQ) that was specially developed for the Lifelines cohort study as an alternative to the regular comprehensive FFQ, and is called the Flower-FFQ [6]. It consists of one main questionnaire (heart-FFQ), which asks for intakes of major food groups, and three short complementary questionnaires (petal FFQs), which ask for detailed information on food types within major food groups of the main questionnaire. The four questionnaires are administered at different time points during a five year period, aiming to reduce participant burden and potentially associated measurement error.

Data on dietary intake in the Lifelines database can, together with other data, be used to investigate associations between diet and diseases. Investigating such associations is complicated because of the complexity of diets: foods and nutrients are consumed in combinations which can induce interactions and synergies between dietary components [7, 8]. Dietary pattern analysis is therefore a useful method to study associations between dietary intake and disease development [9]. One approach to assess dietary patterns is to calculate a dietary index [10, 11], an example of which is the Dutch Healthy Diet index 2015 (DHD2015-index) [12], which assesses adherence to the Dutch dietary guidelines published in 2015 [13, 14], and is a measure of diet quality.

The aim of the current study is to assess the DHD2015-index in the Lifelines cohort, in order to be used by researchers who are investigating diet-disease associations using data from the Lifelines database. Only half of the Lifelines participants completed all the four questionnaires from the Flower-FFQ. In order to evaluate the usefulness of the DHD2015-index score based on data from the heart-FFQ only when data from the petal-FFQs is not available, we also aimed to compare the DHD2015-index based on basic data from the heart-FFQ only with the index based on detailed data from the total Flower-FFQ. This article replaces the retracted article that was published on 16 May 2022 [1].

## Methods

### Study population

Between 2006 and 2013, inhabitants of the northern three provinces of the Netherlands (Friesland, Groningen and Drenthe) and their families, covering three generations, were included in the Lifelines cohort study, with the aim to follow them for at least thirty years. Exclusion criteria included having a severe psychiatric or physical illness, limited life expectancy (<5 years), and insufficient knowledge of the Dutch language to complete a Dutch questionnaire. At baseline, 167,729 participants were included. Every eighteen months, participants complete several questionnaires, including the Flower-FFQ, and every five years, participants undergo physical measurements and biological sampling. A more detailed description of the Lifelines cohort study can be found elsewhere [3, 4].

The Lifelines study is conducted according to the principles of the Declaration of Helsinki and according to the research code of the University Medical Center Groningen (UMCG). The Lifelines study is approved by the medical ethical committee of the UMCG, The Netherlands. All participants gave written informed consent.

### Assessment of dietary intake

Dietary intake was assessed using the Flower-FFQ [6]. Its name is derived from its design: the FFQ consists of one main questionnaire which symbolizes the heart of the flower, and three complementary questionnaires which symbolize the flower petals. The heart-FFQ contains 110 food items used to estimate intakes of major food groups, energy, and macronutrients. The three petal-FFQs ask for detailed information on the types of food consumed within the food groups of the heart-FFQ, as well as supplement intake, to estimate specific (micro)nutrients and food components. Combined, the heart-FFQ and the three petal-FFQs cover 212 food items. A more detailed description of the Flower-FFQ can be found elsewhere [6].

All adult participants of the Lifelines cohort study were invited to complete the Flower-FFQ. During the first assessment (between 2007 and 2013) participants completed the heart-FFQ. During three subsequent assessments (2011–2014, 2012–2015, and 2014–2017) participants completed the petal-FFQs. The petal-FFQs were randomly allocated so that each participant received the petals in one out of six possible orders. Time points were fairly evenly distributed over the years and seasons. These four assessments are referred to as the baseline for dietary intake. At future assessments in the coming years, participants will be invited to complete the heart-FFQ and the petal-FFQs again, which will be referred to as follow-ups for dietary intake.

With data obtained from the total Flower-FFQ and with data obtained from the heart-FFQ only, further referred to as Flower-FFQ and heart-FFQ respectively, the frequency of consumption of food items over the previous month was assessed. Data on food consumption was converted into daily energy and nutrient intake using data from the Dutch food composition database of 2011 [15].

Potential under- or overreporting for the Flower-FFQ and for the heart-FFQ was assessed using Willett’s criteria for implausibly low or high daily energy intake, i.e. <800 and >4200 kcal for men and <500 and >3500 kcal for women [16, 17].

A total of 144,093 adults completed the heart-FFQ, of whom 129,030 participants (90%) reported plausible habitual dietary intake. From participants who completed the heart-FFQ, 68,698 participants completed the total Flower-FFQ, of whom 59,982 participants (87%) reported plausible habitual dietary intake. For 59,881 participants, habitual dietary intake was considered plausible based on both data from the Flower-FFQ and data from the heart-FFQ. Only data from participants with plausible habitual dietary intake is presented.

### Assessment of the DHD2015-index

The DHD2015-index is a measure of adherence to the 2015 Dutch dietary guidelines [12]. The index consists of fifteen components: vegetables, fruits, wholegrain products, legumes, nuts, dairy, fish, tea, fats and oils, coffee, red meat, processed meat, sweetened beverages and fruit juices, alcohol and salt. Recently, the DHD2015-index was further expanded to include a component on unhealthy foods [18], based on a guideline of the Netherlands Nutrition Centre [19]. The present sixteen components can be divided into adequacy, moderation, optimum, qualitative and ratio components. Adequacy components are derived from a guideline that recommends to increase intake (vegetables, fruits, legumes, nuts, fish and tea). Moderation components are derived from a guidelines that recommends to limit intake (red meat, processed meat, sweetened beverages and fruit juices, alcohol, salt and unhealthy food choices). Dairy is an optimum component based on an optimal range of intakes, whereas coffee is a qualitative component based on the type of coffee. The fats and oils component is a ratio component and is based on the ratio of intake of healthy and unhealthy products in that food group. The wholegrain products component is considered as two types of components because two guidelines for grain products exist: an adequacy component for wholegrain intake and a ratio component to reflect replacement of refined grain products by wholegrain products. All components are assigned a score based on intake of the specific food group. To determine the contribution of food items from the FFQ to specific food groups of the DHD2015-index, e.g. wholegrain or refined grains products, for some food items assumptions regarding the percentage contribution of the food item to the food groups had to be made. These assumptions were based on the Dutch National Food Consumption Survey [20]. In case no assumptions could be made, the food item was not used for assessment of the DHD2015-index.

For all components a minimum of 0 points and a maximum of 10 points can be allocated, resulting in a total score ranging from 0 to 160 points, with a higher score indicating better adherence to the guidelines (Table 1). A more detailed description of the DHD2015-index and scoring per component can be found elsewhere [12].

**Table 1:** Components and Dutch dietary guidelines of the DHD15-index and their threshold (minimum score) and cut-off (maximum score).

	Components	Component type	Dutch dietary guidelines 2015	Minimum score (=0)	Maximum score (=10)
1.	Vegetables	A	Eat at least 200 g of vegetables daily.	0 g/day	≥200 g/day
2.	Fruit	A	Eat at least 200 g of fruit daily.	0 g/day	≥200 g/day
3.	Wholegrain products^a^	A	a. Eat at least 90 g of wholegrain products daily.	0 g/day	≥90 g/day
		R	b. Replace refined grain products by wholegrain products.	No consumption of wholegrain products OR ratio wholegrain/refined grains ≤0.7	No consumption of refined grain products OR ratio wholegrain/refined grains ≥11
4.	Legumes	A	Eat legumes weekly.	0 g/day	≥10 g/day
5.	Nuts	A	Eat at least 15 g of unsalted nuts daily.	0 g/day	≥15 g/day
6.	Dairy^b^	O	Eat a few portions of dairy produce daily, including milk or yoghurt.	0 g/day OR ≥750 g/day	300–450 g/day
7.	Fish^c^	A	Eat one serving of fish weekly, preferably oily fish.	0 g/day	≥15 g/day
8.	Tea	A	Drink three cups of black or green tea daily.	0 g/day	≥450 g/day
9.	Fats and oils	R	Replace butter, hard margarines, and cooking fats by soft margarines, liquid cooking fats, and vegetable oils.	No consumption of soft margarines, liquid cooking fats, and vegetable oils OR ratio liquid cooking fats/solid cooking fats ≤0.6	No consumption of butter, hard margarines, and cooking fats OR ratio liquid cooking fats/solid cooking fats ≥13
10.	Coffee	Q	Replace unfiltered coffee by filtered coffee.	Any consumption of unfiltered coffee	Consumption of only filtered coffee OR no coffee consumption
11.	Red meat	M	Limit consumption of red meat.	≥100 g/day	≤45 g/day
12.	Processed meat	M	Limit consumption of processed meat.	≥50 g/day	0 g/day
13.	Sweetened beverages and fruit juices	M	Limit consumption of sweetened beverages and fruit juices.	≥250 g/day	0 g/day
14.	Alcohol	M	If alcohol is consumed at all, intake should be limited to one Dutch units (10 g ethanol) daily.	Women: ≥ 20 g ethanol/day Men: ≥30 g ethanol/day	Women: ≤10 g ethanol/day Men: ≤10 g ethanol/day
15.	Salt	M	Limit consumption of table salt to 6 g daily.	≥3.8 g sodium/day	≤1.9 g sodium/day
16.	Unhealthy choices	M	Limit consumption of unhealthy choices	>7 choices/week	≤3 choices/week

*A* adequacy component, *M* moderation component, *O* optimum component, *Q* qualitative component, *R* ratio component.

^a^This component consists of two subcomponents (a and b). Each subcomponent has a maximum score of 5 points.

^b^Maximum of 40 g cheese can be included.

^c^Maximum of 4 g lean fish can be included.

The DHD2015-index was assessed with data from the Flower-FFQ and with data from the heart-FFQ. From the Flower-FFQ, data on filtering of coffee and salt intake is not available, so these two components were not included in the DHD2015-index calculations. From the heart-FFQ, regarding the wholegrain products component, only the adequacy component, and not the ratio component, with a maximum of 5 points can be assessed. This results in total scores ranging from 0 to 140 points for the DHD2015-index from the Flower-FFQ, and 0 to 135 points for the DHD2015-index from the heart-FFQ.

### Assessment of other characteristics

Data on sex, age, socioeconomic status (SES), smoking, and physical activity were obtained from questionnaires. SES was categorized based on education attainment [21], as follows: no education, primary education, lower vocational education, lower general secondary education (low); intermediate vocational education, higher general secondary education (moderate); higher vocational education and university education (high). Smoking was categorized as current, former and never smoker. Physical activity was assessed with the short questionnaire to assess health-enhancing physical activity [22], from which the average number of minutes per week of various domains of physical activity were assessed. Metabolic equivalent of task (MET) values were assigned to the specific physical activities [23], and the total number of minutes per week of moderate to vigorous physical activity (MVPA) was calculated, using MET values of ≥4.0 to <6.5 for moderate physical activity and MET values ≥ 6.5 for vigorous physical activity.

Anthropometric measurements, including height and weight, were conducted by well-trained staff at Lifelines research facilities. Body mass index (BMI) was calculated as kg/m^2^.

### Statistical analyses

Data were checked for normality using a Kolmogorov–Smirnov test and visual inspection of Q-Q normality plots. All continuous variables, except the DHD2015-index total scores from both the Flower-FFQ and the heart-FFQ, showed a skewed distribution and are therefore presented as medians with 25th–75th percentiles. Categorical variables are presented as numbers with percentages.

The DHD2015-index and the component scores were compared between men and women using a Mann–Whitney U test. Trends in participants’ characteristics and energy and nutrient intake across quartiles of the DHD2015-index were examined using a Jonckheere-Terpstra test. These analyses were performed with both data from the Flower-FFQ and data from the heart-FFQ.

To compare the DHD2015-index from the Flower-FFQ and from the heart-FFQ regarding ranking of participants, Kendall’s tau-b correlation coefficients (*r*) were calculated between total scores and component scores, and classified as good (*r* ≥ 0.50), acceptable (*r* 0.20–0.49), or poor (*r* < 0.20) [24]. Confidence intervals were calculated using a Fisher’s z-transformation. Agreement between the DHD2015-index from the Flower-FFQ and from the heart-FFQ was examined with a Bland–Altman plot [25], and with cross-classification into quartiles of the DHD2015-index, for which a good outcome was considered if more than 50% of participants were classified in the same quartile [24].

The level of significance for all statistical tests was set at *p* < 0.05. Statistical analyses were performed with SPSS software (Version 25, IBM, Armonk, NY, USA).

## Results

### Participant characteristics

Table 2 presents characteristics of participants who completed the Flower-FFQ, and of participants who completed the heart-FFQ, regardless of whether they completed all three petal-FFQs as well. Among participants who completed the Flower-FFQ, 40% were men. The median (25th–75th percentile) age was 47 (36–56) for men and 46 (38–54) for women. Among participants who completed the heart-FFQ, 41% were men, and the median (25th–75th percentile) age was 45 (36–54) for men and 44 (35–52) for women. Differences in characteristics between participants who completed and did not complete the total Flower-FFQ are described elsewhere [5].

**Table 2:** Characteristics of participants who completed the Flower-FFQ (*n* = 59,982) and who completed the heart-FFQ (*n* = 129,030).

	Flower-FFQ (*n* = 59,982)				Heart-FFQ (*n* = 129,030)			
	Men (*n* = 23,703)		Women (*n* = 36,279)		Men (*n* = 53,137)		Women (*n* = 75,893)	
	Median/*n*	25th–75th percentile/%	Median/*n*	25th–75th percentile/%	Median/*n*	25th–75th percentile/%	Median/*n*	25th–75th percentile/%
Age (years)	47	39–56	46	38–54	45	36–54	44	35–52
SES								
Low	6590	27.8	10,500	28.9	15,137	28.5	21,989	29.0
Moderate	8563	36.1	14,314	39.5	19,935	37.5	30,499	40.2
High	8143	34.4	10,861	29.9	16,944	31.9	21,921	28.9
Unknown	407	1.7	604	1.7	1121	2.1	1484	2.0
Smoking								
Current smoker	4468	18.8	5876	16.2	11,919	22.4	14,604	19.2
Former smoker	8722	36.8	12,480	34.4	17,964	33.8	24,231	31.9
Never smoker	10,359	43.7	17,681	48.7	22,859	43.0	36,473	48.1
Unknown	154	0.6	242	0.7	395	0.7	585	0.8
Physical activity: MVPA (minutes/week)	285	120–627	245	115–520	281	105–630	240	90–504
BMI (kg/m^2^)	25.9	23.9–28.2	24.8	22.5–27.9	26.0	23.9–28.4	24.9	22.5–28.1

*SES* socioeconomic status, *MVPA* moderate to vigorous physical activity, *BMI* body mass index.

### DHD2015-index scores

The DHD2015-index scores were higher for women than for men (Table 3). The median (25th–75th percentile) DHD2015-index score from the flower-FFQ was 75 (65–85) for men and 81 (70–91) for women; based on the heart-FFQ these values were 68 (58–77) for men and 73 (63–82) for women. Generally, the highest component scores were obtained for the components red meat and alcohol, and the lowest scores for the component unhealthy choices, both in men and women. Women scored higher than men on vegetables, fruit, dairy, tea, processed meat, and sweetened beverages and fruit juices, based on both the Flower-FFQ and the heart-FFQ, and higher on fats and oils based on only the Flower-FFQ. Men scored higher than women on legumes, nuts and fish, based on both the Flower-FFQ and the heart-FFQ. Men also scored higher on -wholegrain products intake, based on only the heart-FFQ.

**Table 3:** DHD2015-index score and it component scores based on the Flower-FFQ (*n* = 59,982) and based on the heart-FFQ (*n* = 129,030).

	Flower-FFQ (*n* = 59,982)					Heart-FFQ (*n* = 129,030)				
	Men (*n* = 23,703)		Women (n = 36,279)		*p*-value^a^	Men (*n* = 53,137)		Women (*n* = 75,893)		*p*-value^a^
	Median	25th–75th percentile	Median	25th–75th percentile	Between sexes	Median	25th–75th percentile	Median	25th–75th percentile	Between sexes
DHD2015-index score	75.0	64.8–85.4	80.5	70.2–91.0	<0.001	67.6	57.8–77.3	72.6	63.0–82.4	<0.001
DHD2015-index components										
1. Vegetables	6.6	4.5–9.2	7.4	5.3–10.0	<0.001	4.1	3.1–5.5	5.3	3.7–7.4	<0.001
2. Fruit	4.9	2.0–10.0	6.4	3.3–10.0	<0.001	4.2	2.1–10.0	5.5	2.1–10.0	<0.001
3a. Wholegrain products intake	5.0	5.0–5.0	5.0	4.3–5.0	<0.001	3.2	2.4–4.1	2.4	1.8–3.0	<0.001
3b. Ratio wholegrain/refined grains	1.5	0.6–4.2	1.5	0.6–4.7	0.001					
3. Wholegrain products total^b^	6.4	5.5–9.0	6.3	5.3–8.9	<0.001					
4. Legumes	6.6	0.0–10.0	4.4	0.0–10.0	<0.001	6.6	0.0–10.0	4.4	0.0–10.0	<0.001
5. Nuts	4.6	1.6–9.2	3.4	1.0–7.0	<0.001	4.6	1.4–9.2	3.4	0.9–6.5	<0.001
6. Dairy	8.1	5.3–10.0	8.2	5.5–10.0	<0.001	7.9	5.0–10.0	8.0	5.2–10.0	<0.001
7. Fish	4.8	2.7–8.0	4.4	1.4–7.6	<0.001	4.1	2.7–6.4	3.8	2.7–6.4	0.001
8. Tea	1.8	0.1–5.2	3.6	1.1–6.9	<0.001	2.0	0.2–5.2	5.2	2.0–10.0	<0.001
9. Fats and oils	6.6	0.2–10.0	10.0	0.4–10.0	<0.001	1.7	0.0–10.0	1.8	0.0–10.0	0.472
11. Red meat	10.0	10.0–10.0	10.0	10.0–10.0	<0.001	10.0	9.9–10.0	10.0	10.0–10.0	<0.001
12. Processed meat	2.3	0.0–5.1	4.9	2.3–7.1	<0.001	2.4	0.0–5.1	4.8	2.3–6.9	<0.001
13. Sweetened beverages and fruit juices	4.4	0.0–8.0	6.1	1.7–8.9	<0.001	3.9	0.0–7.8	5.7	0.8–8.7	<0.001
14. Alcohol	10.0	7.3–10.0	10.0	10.0–10.0	<0.001	10.0	7.1–10.0	10.0	10.0–10.0	<0.001
16. Unhealthy choices	0.0	0.0–0.0	0.0	0.0–0.0	<0.001	0.0	0.0–0.0	0.0	0.0–0.0	<0.001

^a^*P*-values are obtained with a Mann–Whitney U test. Note that because of the large study population, even small differences that are not visible with rounding to 1 decimal, turned out to be statistically significant, which may not always be relevant differences.

^b^Sum score of components 3a and 3b.

The DHD2015-index score from the Flower-FFQ was positively associated with age, SES, physical activity, and intake of protein, dietary fiber, and micronutrients, both in men and women (Table 4). Inverse associations were observed for smoking, and intake of energy, carbohydrate and fat, both in men and women. For the DHD2015-index score from the heart-FFQ, similar associations were observed (Supplementary Table 1).

**Table 4:** Participant characteristics across quartiles of the DHD2015-index based on the Flower-FFQ (*n* = 59,982).

	Quartiles DHD2015-index based on the Flower-FFQ								
	Q1		Q2		Q3		Q4		
	Median/*n*	25th–75th percentile/%	Median/*n*	25th–75th percentile/%	Median/*n*	25th–75th percentile/%	Median/*n*	25th–75th percentile/%	*p*-value for trend^a^
Men (*n* = 23,703)	*n* = 5925		*n* = 5926		*n* = 5926		*n* = 5926		
DHD2015-index score	57.8	51.9–61.7	70.2	67.6–72.7	80.0	77.5–82.6	92.6	88.7–98.3	
Age (years)	43	33–49	46	38–53	48	41–57	53	45–62	<0.001
SES									<0.001
Low	1884	31.8	1623	27.4	1575	26.6	1508	25.4	
Moderate	2436	41.1	2277	38.4	2045	34.5	1805	30.5	
High	1512	25.5	1935	32.7	2206	37.2	2490	42.0	
Unknown	93	1.6	91	1.5	100	1.7	123	2.1	
Smoking									<0.001
Current smoker	1722	29.1	1220	20.6	924	15.6	602	10.2	
Former smoker	1654	27.9	2005	33.8	2368	40.0	2695	45.5	
Never smoker	2514	42.4	2660	44.9	2600	43.9	2585	43.6	
Unknown	35	0.6	41	0.7	34	0.6	44	0.7	
Physical activity: MVPA (minutes/week)	270	75 - 720	270	100–620	274	120–589	315	150–600	<0.001
BMI (kg/m^2^)	25.9	23.8–28.2	26.0	24.0–28.2	26.0	24.1–28.3	25.8	23.9–28.1	0.995
Energy intake (kcal/day)	2462	2053–2950	2401	1993–2853	2345	1973–2773	2273	1893–2687	<0.001
Total carbohydrate intake									
g/day	264	214–320	258	210–312	253	208–305	249	204–297	<0.001
En%	45.1	41.2–49.0	45.2	41.4–49.0	45.3	41.7–49.1	45.7	42.1–49.5	<0.001
Total fat intake									
g/day	101	80–128	98	78–122	94	75–118	88	69–110	<0.001
En%	37.8	33.3–42.8	37.2	33.0–42.1	36.7	32.4–41.3	35.3	31.2–40.0	<0.001
Total protein intake									
g/day	83	69–98	84	71–98	84	72–98	85	73–98	<0.001
En%	14.1	12.8–15.6	14.7	13.4–16.1	15.1	13.8–16.5	15.7	14.4–17.1	<0.001
Fiber									
(g)	22	17–27	24	20–29	25	21–30	28	23–33	<0.001
(En%)	1.7	1.5–1.9	1.9	1.7–2.2	2.1	1.9–2.3	2.3	2.1–2.6	<0.001
Retinol equivalents (µg)	1108	806–1533	1148	866–1549	1168	887–1561	1164	905–1523	<0.001
Vitamin B2 (mg)	1.5	1.2–1.9	1.5	1.2–1.8	1.5	1.2–1.8	1.5	1.3–1.8	<0.001
Vitamin B6 (mg)	1.5	1.2–1.8	1.5	1.2–1.8	1.5	1.3–1.8	1.6	1.3–1.8	<0.001
Folate (present in food by nature) (µg)	225	185–275	244	203–290	259	217–305	285	242–334	<0.001
Folate equivalents (µg)	230	188–282	250	208–302	268	223–322	296	249–356	<0.001
Vitamin B12 (µg)	3.8	2.9–5.2	3.9	3.0–5.3	4.0	3.1–5.4	4.2	3.2–5.6	<0.001
Vitamin C (mg)	73	53–100	86	63–112	96	72–126	114	86–145	<0.001
Vitamin E (mg)	12	10–16	13	10–17	14	11–18	14	11–18	<0.001
Calcium (mg)	924	698–1228	969	774–1216	993	805–1230	1043	860–1261	<0.001
Women (*n* = 32,279)	*n* = 9069		*n* = 9071		*n* = 9069		*n* = 9070		
DHD2015-index score	63.1	57.6–67.0	75.7	73.1–78.2	85.5	82.9–88.1	98.2	94.3–103.8	
Age (years)	42	32–48	45	37–51	47	40–55	51	44-60	<0.001
SES									<0.001
Low	2716	29.9	2576	28.4	2578	28.4	2630	29.0	
Moderate	4044	44.6	3756	41.4	3462	38.2	3052	33.6	
High	2176	24.0	2589	28.5	2886	31.8	3210	35.4	
Unknown	133	1.5	150	1.7	143	1.6	178	2.0	
Smoking									<0.001
Current smoker	2430	26.8	1508	16.6	1129	12.4	809	8.9	
Former smoker	2405	26.5	2955	32.6	3339	36.8	3781	41.7	
Never smoker	4182	46.1	4551	50.2	4537	50.0	4411	48.6	
Unknown	52	0.6	57	0.6	64	0.7	69	0.8	
Physical activity: MVPA (minutes/week)	215	70–510	240	105–487	256	120–520	300	140–540	<0.001
BMI (kg/m^2^)	24.8	22.4–27.9	24.9	22.6–28.0	24.9	22.6–28.0	24.8	22.6–27.6	0.784
Energy intake (kcal/day)	1888	1585–2226	1859	1562–2197	1843	1548–2165	1805	1506–2121	<0.001
Total carbohydrate intake									
g/day	206	170–248	204	167–244	203	168–240	198	164–236	<0.001
En%	45.8	41.7–49.7	45.5	41.8–49.1	45.6	42.0–49.2	45.7	42.1–49.4	0.650
Total fat intake									
g/day	77	61–96	74	59–93	72	57–89	68	53–85	<0.001
En%	37.1	32.9–41.7	36.1	32.3–40.6	35.3	31.4–39.5	34.2	30.3–38.4	<0.001
Total protein intake									
g/day	67	57–79	70	60–81	72	61–82	73	63–84	<0.001
En%	14.9	13.4–16.4	15.5	14.1–17.1	16.0	14.6–17.6	16.8	15.2–18.5	<0.001
Fiber									
(g)	18	15–21	20	17–24	22	18–26	24	20–28	<0.001
(En%)	1.8	1.6–2.1	2.1	1.8–2.3	2.3	2.0–2.6	2.5	2.3–2.9	<0.001
Retinol equivalents (µg)	895	673–1187	937	720–1216	973	768–1235	1018	816–1278	<0.001
Vitamin B2 (mg)	1.2	1.0–1.5	1.3	1.0–1.5	1.3	1.1–1.6	1.4	1.1–1.6	<0.001
Vitamin B6 (mg)	1.2	1.0–1.5	1.3	1.1–1.5	1.3	1.1–1.6	1.4	1.2–1.6	<0.001
Folate (present in food by nature) (µg)	193	159–229	217	184–254	237	204–276	267	231–309	<0.001
Folate equivalents (µg)	196	161–234	221	187–262	243	207–287	275	237–324	<0.001
Vitamin B12 (µg)	3.2	2.4–4.2	3.3	2.6–4.3	3.5	2.7–4.5	3.7	2.9–5.0	<0.001
Vitamin C (mg)	74	53–99	88	65–117	102	77–131	119	96–148	<0.001
Vitamin E (mg)	10	8–13	11	9–14	11	9–14	12	9–14	<0.001
Calcium (mg)	828	638–1064	891	719–1098	938	761–1144	1001	826–1206	<0.001

*SES* socioeconomic status, *MVPA* moderate to vigorous physical activity, *BMI* body mass index.

^a^*P*-values are obtained with a Jonckheere–Terpstra test.

### Comparison of DHD2015-index scores between the Flower-FFQ and the heart-FFQ

The median DHD2015-index scores from the Flower-FFQ were higher than the median scores from the heart-FFQ; the difference in median was 5.8 points for men and 6.6 points for women (Table 5). When the ratio component for grains was not included in the DHD2015-index score from the Flower-FFQ, the difference in median scores was 3.8 points for men and 4.5 point for women. Component scores from the Flower-FFQ were higher than scores from the heart-FFQ for vegetables, whole grain products intake, fish, and fats and oils, and lower for fruit and tea, both in men and women. Differences in median component scores were small, except for fats and oils, for which the difference was 4.8 points in men and 8.2 points in women.

**Table 5:** Comparison of DHD2015-index and it component scores based on the Flower-FFQ and based on the heart-FFQ (*n* = 59,881).

	Men (*n* = 23,646)						Women (*n* = 36,235)					
	Flower-FFQ		Heart-FFQ				Flower-FFQ		Heart-FFQ			
	Median	25th–75th percentile	Median	25th–75th percentile	*r*^a^	95% CI	Median	25th–75th percentile	Median	25th–75th percentile	*r*^a^	95% CI
DHD2015-index score	75.0	64.8–85.4	69.2	59.6–78.8	0.67	0.66–0.68	80.5	70.2–91.0	73.9	64.4–83.4	0.66	0.66–0.67
DHD2015-index score (adjusted)^b^	73.0	62.9–83.0	69.2	59.6–78.8	0.67	0.66–0.68	78.4	68.4–88.5	73.9	64.4–83.4	0.67	0.67–0.68
DHD2015-index components												
1. Vegetables	6.6	4.5–9.2	5.3	3.1–6.1	0.69	0.68–0.70	7.4	5.3–10.0	5.3	3.7–7.4	0.66	0.66–0.67
2. Fruit	5.0	2.0–10.0	5.5	2.1–10.0	0.93	0.93–0.93	6.5	3.3–10.0	7.6	3.8–10.0	0.91	0.91–0.91
3a. Wholegrain products intake	5.0	5.0–5.0	3.2	2.5–4.1	0.38	0.37–0.39	5.0	4.3–5.0	2.4	1.8–3.0	0.50	0.49–0.51
3b. Ratio wholegrain/refined grains	1.5	0.6–4.2					1.5	0.6–4.7				
3. Wholegrain products total^c^	6.4	5.5–9.0					6.3	5.3–8.9				
4. Legumes	6.6	0.0–10.0	6.6	0.0–10.0	1.00	1.00–1.00	4.4	0.0–10.0	4.4	0.0–10.0	1.00	1.00–1.00
5. Nuts	4.6	1.6–9.2	4.6	1.6–9.2	1.00	1.00–1.00	3.4	1.0–7.0	3.4	1.0–7.0	1.00	1.00–1.00
6. Dairy	8.1	5.3–10.0	8.1	5.3–10.0	0.94	0.94–0.94	8.2	5.5–10.0	8.2	5.5–10.0	0.95	0.95–0.95
7. Fish	4.8	2.7–8.0	4.2	2.7–6.5	0.63	0.62–0.64	4.4	1.4–7.6	4.1	2.7–6.5	0.65	0.65–0.66
8. Tea	1.8	0.1–5.2	2.6	0.4–5.2	0.81	0.81–0.81	3.6	1.1–6.9	5.2	2.6–10.0	0.73	0.73–0.73
9. Fats and oils	6.6	0.2–10.0	1.8	0.0–10.0	0.16	0.15–0.17	10.0	0.4–10.0	1.8	0.0–10.0	0.16	0.15–0.17
11. Red meat	10.0	10.0–10.0	10.0	9.9–10.0	0.60	0.59–0.61	10.0	10.0–10.0	10.0	10.0–10.0	0.57	0.56–0.58
12. Processed meat	2.3	0.0–5.1	2.5	0.0–5.2	0.75	0.75–0.76	4.9	2.3–7.1	4.8	2.4–6.9	0.70	0.70–0.71
13. Sweetened beverages and fruit juices	4.4	0.0–8.0	4.4	0.0–7.9	0.95	0.95–0.95	6.1	1.7–8.9	5.8	1.5–8.9	0.94	0.94–0.94
14. Alcohol	10.0	7.3–10.0	10.0	7.3–10.0	1.00	1.00–1.00	10.0	10.0–10.0	10.0	10.0–10.0	1.00	1.00–1.00
16. Unhealthy choices	0.0	0.0–0.0	0.0	0.0–0.0	0.72	0.71–0.73	0.0	0.0–0.0	0.0	0.0–0.0	0.76	0.76–0.76

*CI* confidence interval.

^a^Kendall’s tau-b correlation coefficient.

^b^For calculation of the DHD2015-index score based on the heart-FFQ, component 3b is not included. Therefore, we also calculated the DHD2015-index score based on the Flower-FFQ without component 3b.

^c^Sum score of components 3a and 3b.

**Fig. 1:**
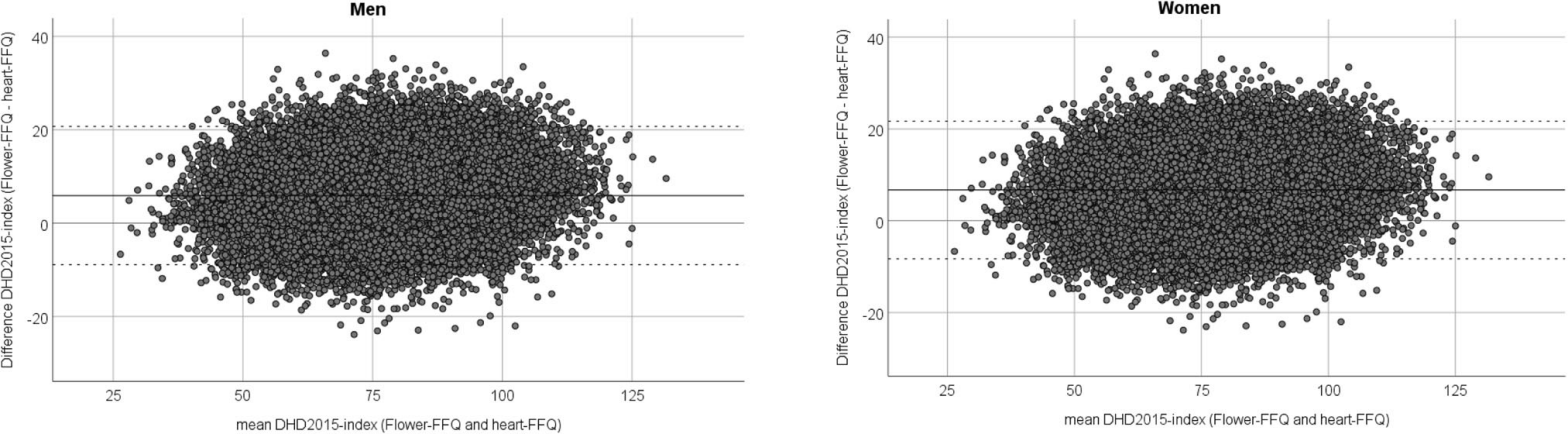
Bland–Altman plots for the DHD2015-index score from the Flower-FFQ and from the heart-FFQ. Bland–Altman plots for the DHD2015-index score from the Flower-FFQ and from the heart-FFQ in men (left) and women (right).

The Kendall’s tau-b correlation coefficient between the two DHD2015-index scores was 0.67 (95% confidence interval (CI) 0.66–0.68) for men and 0.66 (95% CI 0.66–0.67) for women. Between the component scores, it ranged from 0.16 (95% CI 0.15–0.17) for fats and oils to 1.00 (95% CI 1.00–1.00) for legumes, nuts and alcohol, in both men and women. Agreement between the two DHD2015-index scores is graphically presented in a Bland–Altman plot (Fig. 1). In men, the mean difference between the DHD2015-index from the Flower-FFQ and the heart-FFQ was 5.9 points and the limits of agreement were −8.9 and 20.7 points. In women, the mean difference was 6.7 points and the limits of agreement were −8.3 and 21.7 points.

Results from cross-classification into quartiles of the DHD2015-index showed that 59% of men was classified in the same quartile, 37% in the adjacent quartile, and 4% in the non-adjacent quartile. For women, these percentages were 60%, 36%, and 4% respectively.

## Discussion

We assessed the DHD2015-index in the Lifelines cohort, based on data from the total Flower-FFQ and based on data from the heart-FFQ only. The indices from the Flower-FFQ and from the heart-FFQ showed good agreement of ranking participants according to diet quality, although differences were observed for certain component scores.

The DHD2015-index scores were higher for women than for men (median differences were 5.5 and 5.0 points for scores from the Flower-FFQ and from the heart-FFQ, respectively), which can be explained by better adherence to the dietary guidelines, particularly to the guidelines for intake of vegetables, fruit, dairy, tea, processed meat, and sweetened beverages and fruit juices. Several studies have shown that women have a better diet quality than men [26] and other studies in which the DHD2015-index was assessed using 24 h dietary recalls, a regular FFQ, and a short FFQ specifically developed to assess the DHD2015-index, also reported a higher DHD2015-index for women than for men [12, 18].

In general, the DHD2015-index score was higher in participants who were older, had a higher SES, and were more physically active, whilst the index was lower in smoking participants. These findings are in agreement with the literature [12, 18, 26]. These studies also found an inverse association with BMI, but we did not observe an association with BMI. One explanation for this may be that misreporting is more common among participants with a high BMI [27], which can mask the true association. Another explanation may be that these participants adhere more closely to the dietary guidelines in response to their high BMI, in an effort to lose weight and improve their health [5]. Regarding nutrient intake, the DHD2015-index was positively associated with intake of protein, dietary fiber and micronutrients, and inversely associated with intake of energy, carbohydrate and fat, which indeed indicates a healthier diet. These associations of the DHD2015-index with energy and nutrient intake were also observed in a previous study [12]. It should be noted that because of the large study population, even small differences and associations turned out to be statistically significant, which may not always be relevant differences.

Median DHD2015-index scores from the Flower-FFQ and from the heart-FFQ were comparable and showed good correlation and cross-classification into quartiles, indicating good agreement of ranking participants according diet quality. Despite good agreement for the total scores, certain component scores differed. Although most correlation coefficients were classified as good, the component score for fats and oils showed poor correlation in both men and women. The component score for wholegrain products intake was acceptable in men and just within the range to be classified as good in women. This may be explained by a difference in the degree of detail requested in the Flower-FFQ and the heart-FFQ. For example, the heart-FFQ provides basic information about the total amount of bread consumed crudely, without distinguishing bread type. More detailed information about bread type is provided by the third petal-FFQ. To assess the score for wholegrain products based on the heart-FFQ, assumptions were made regarding the percentages of wholegrain and refined grains products, and this was also true for other components. Fewer assumptions, however, had to be made for scores based on the Flower-FFQ, meaning the DHD2015-index based on the Flower-FFQ gives a better reflection of diet quality than the index based on the heart-FFQ.

A strength of the Lifelines cohort study is the large study population. A limitation of this study is the self-reporting method using an FFQ for dietary intake assessment. All self-reporting methods are prone to several types of error such as recall bias or the tendency to provide socially desirable answers [28]. An FFQ may be time-consuming and therefore considered burdensome to complete, which may result in biased answers. The Flower-FFQ was especially developed for the Lifelines cohort study as an alternative to a regular FFQ consisting of one comprehensive questionnaire. As the Flower-FFQ consists of four questionnaires that are administered at different time points during a five year period, experienced burden and risk of bias may be lower for this FFQ than for a regular FFQ. A disadvantage is that changes in dietary intake may occur within the five years, although stable food consumption patterns over time are assumed [29]. Furthermore, an FFQ is not the best method to evaluate absolute intake of foods and nutrients, however, it is a reliable method to rank participants to their intake levels [30, 31], and consequently, to rank participants to diet quality. In epidemiologic studies on associations between diet and diseases, such as the Lifelines cohort study, ranking of participants according to their intake levels or diet quality is usually more relevant than evaluating absolute levels of intake or quality measures.

## Conclusion

The DHD2015-index assesses adherence to the 2015 Dutch dietary guidelines and is a measure of diet quality. We assessed the DHD2015-index in the Lifelines cohort, and this index can be used by researchers who are investigating diet-disease associations using data from the Lifelines database. The DHD2015-index was assessed with data from the Flower-FFQ and with data from the heart-FFQ. The Flower-FFQ asks for more detailed information on dietary intake and provides more optimal information than the heart-FFQ to assess the DHD2015-index. Therefore, the DHD2015-index from the Flower-FFQ should be preferred. However, the DHD2015-index from the heart-FFQ showed good agreement with the index from the Flower-FFQ of ranking participants according to diet quality, and can therefore be used when the index from the Flower-FFQ is not available, although for some components the heart-FFQ provides limited information.

### Supplementary information

Supplementary table 1

## Data Availability

Data may be obtained from a third party and are not publicly available. Researchers can apply to use the Lifelines data used in this study. More information about how to request Lifelines data and the conditions of use can be found on their website (https://www.lifelines.nl/researcher/how-to-apply).
