# Supplementary material for: Assessment of the Dutch Healthy Diet index 2015 in the Lifelines cohort study at baseline
Source: Eur J Clin Nutr. 2023 Nov 28;78(3):217–27. doi: 10.1038/s41430-023-01372-x (PMC10927538; doi:10.1038/s41430-023-01372-x)
Supplement: Supplementary file 1 — Supplementary table 1 [file 41430_2023_1372_MOESM1_ESM.docx]

**Supplementary table 1.** Participant characteristics across quartiles of the DHD2015-index based on the heart-FFQ (n=129,030)

|  | **Quartiles DHD2015-index based on the heart-FFQ** | | | | | | | |  |
| --- | --- | --- | --- | --- | --- | --- | --- | --- | --- |
|  | **Q1** | | **Q2** | | **Q3** | | **Q4** | |  |
|  | **median / n** | **25^th^ – 75^th^ percentile / %** | **median / n** | **25^th^ – 75^th^ percentile / %** | **median / n** | **25^th^ – 75^th^ percentile / %** | **median / n** | **25^th^ – 75^th^ percentile / %** | **p-value for trend*** |
| **Men (n=53,173)** | **n=13,284** | | **n=13,284** | | **n=13,285** | | **n=13,284** | |  |
| DHD2015-index score | 51.2 | 46.0 – 54.8 | 62.8 | 60.4 – 65.2 | 72.2 | 69.8 – 74.6 | 84.2 | 80.4 – 89.6 |  |
| Age (years) | 41 | 31 – 48 | 44 | 35 – 51 | 47 | 38 – 56 | 50 | 42 – 61 | <0.001 |
| SES  Low  Moderate  High  Unknown | 4318 5580 3114 272 | 32.5 42.0 23.4 2.0 | 3857 5177 3974 276 | 29.0 39.0 29.9 2.1 | 3660 4960 4379 286 | 27.5 37.3 33.0 2.2 | 3302 4218 5477 287 | 24.9 31.8 41.2 2.2 | <0.001 |
| Smoking  Current smoker  Former smoker  Never smoker  Unknown | 4585 3323 5278 98 | 34.5 25.0 39.7 0.7 | 3286 4153 5750 95 | 24.7 31.2 43.3 0.7 | 2442 4852 5886 105 | 18.4 36.5 44.3 0.8 | 1606 5636 5945 97 | 12.1 42.4 44.8 0.7 | <0.001 |
| Physical activity: MVPA (minutes/week) | 250 | 60 – 702 | 270 | 90 – 630 | 275 | 120 – 600 | 315 | 150 – 600 | <0.001 |
| BMI (kg/m^2^) | 26.0 | 23.8 – 28.5 | 26.2 | 24.1 – 28.6 | 26.1 | 24.1 – 28.4 | 25.7 | 23.7 – 27.9 | <0.001 |
| Energy intake (kcal/day) | 2354 | 1932 – 2823 | 2299 | 1908 – 2743 | 2288 | 1905 – 2721 | 2266 | 1900 – 2660 | <0.001 |
| Total carbohydrate intake  g/day  En% | 257 44.6 | 207 – 314 40.8 – 48.3 | 254 45.0 | 206 – 309 41.4 – 48.6 | 255 45.4 | 209 – 308  42.0 – 48.8 | 257 46.0 | 212 – 306 42.8 – 49.4 | 0.817 <0.001 |
| Total fat intake  g/day  En% | 93 35.3 | 73 – 116 31.9 – 38.6 | 91 35.2 | 73 – 112 32.0 – 38.3 | 90 34.8 | 72 – 111 31.7 – 37.9 | 87 33.9 | 69 – 106 30.9 – 37.0 | <0.001 <0.001 |
| Total protein intake  g/day  En% | 81 14.0 | 67 – 97 12.7 – 15.4 | 82 14.4 | 69 – 96 13.2 – 15.8 | 83 14.7 | 70 – 96 13.5 – 16.0 | 83 14.9 | 71 – 96 13.8 – 16.2 | <0.001 <0.001 |
| **Women (n=75,893)** | **n=18,973** | | **n=18,974** | | **n=18,973** | | **n=18,973** | |  |
| DHD2015-index score | 56.5 | 51.3 – 60.1 | 68.1 | 65.7 – 70.4 | 77.3 | 74.9 – 79.7 | 89.2 | 85.4 – 94.7 |  |
| Age (years) | 40 | 30 – 48 | 43 | 34 – 50 | 46 | 37 – 54 | 49 | 41 – 58 | <0.001 |
| SES  Low  Moderate  High  Unknown | 5980 8539 4004 350 | 31.5 45.0 21.6 1.8 | 5451 8085 5076 362 | 28.7 42.6 26.8 1.9 | 5418 7472 5727 365 | 28.6 39.4 30.2 1.9 | 5140 6403 7014 416 | 27.1 33.7 37.0 2.2 | <0.001 |
| Smoking  Current smoker  Former smoker  Never smoker  Unknown | 6035 4793 8015 130 | 31.8 25.3 42.2 0.7 | 3847 5753 9213 161 | 20.3 30.3 48.6 0.8 | 2778 6393 9671 131 | 14.6 33.7 51.0 0.7 | 1944 7292 9574 163 | 10.2 38.4 50.5 0.9 | <0.001 |
| Physical activity: MVPA (minutes/week) | 200 | 60 – 500 | 238 | 90 – 480 | 240 | 110 – 495 | 270 | 130 – 521 | <0.001 |
| BMI (kg/m^2^) | 24.9 | 22.4 – 28.5 | 25.0 | 22.6 – 28.3 | 25.0 | 22.7 – 28.2 | 24.7 | 22.4 – 27.6 | <0.001 |
| Energy intake (kcal/day) | 1802 | 1506 – 2134 | 1795 | 1508 – 2118 | 1795 | 1507 – 2110 | 1772 | 1486 – 2081 | <0.001 |
| Total carbohydrate intake  g/day  En% | 201 45.4 | 164 – 243 41.6 – 49.3 | 201 45.6 | 166 – 241 42.1 – 49.1 | 202 45.9 | 168 – 240 42.6 – 49.2 | 202 46.3 | 168 – 239 43.0 – 50.0 | 0.455 <0.001 |
| Total fat intake  g/day  En% | 72 35.5 | 58 – 89 32.2 – 38.7 | 71 35.0 | 57 – 87 31.9 – 38.1 | 69 34.4 | 56 – 86 31.2 – 37.4 | 67 33.4 | 53 – 83 30.1 – 36.4 | <0.001 <0.001 |
| Total protein intake  g/day  En% | 65 14.6 | 54 – 76 13.2 – 16.2 | 67 15.1 | 57 – 78 13.7 – 16.6 | 68 15.4 | 58 – 79 14.1 – 16.8 | 69 15.7 | 59 – 80 14.4 – 17.3 | <0.001 <0.001 |

Abbreviations: SES, socioeconomic status; MVPA, moderate to vigorous physical activity; BMI, body mass index.

* P-values are obtained with a Jonckheere-Terpstra test.
